# Risk factors for postoperative complications after pheochromocytoma and/or paraganglioma: a single-center retrospective study

**DOI:** 10.3389/fonc.2023.1174836

**Published:** 2023-05-05

**Authors:** Lulu Ma, Xuerong Yu, Yuguang Huang

**Affiliations:** Department of Anesthesiology, Peking Union Medical College Hospital, Beijing, China

**Keywords:** pheochromocytoma, paraganglioma, postoperative complication, laparoscopy, laparotomy

## Abstract

**Background:**

Perioperative complications are higher in patients with pheochromocytoma and/or paraganglioma. The aim of this study was to identify the risk factors of postoperative complications after pheochromocytoma and/or paraganglioma resection surgery.

**Method:**

We retrospectively reviewed 438 patients who underwent laparoscopic or open surgery for pheochromocytoma and/or paraganglioma at our center between January 2014 and December 2019. Demographic characteristics and intraoperative and postoperative data were recorded. Complications were defined as deviations from the normal postoperative course and Clavien–Dindo classification was used to grade the severity of postoperative complication. Patients with complications of grade II or above were included for analysis. Binary logistic regression was used to determine the risk factors for postoperative complications.

**Results:**

The median age of the patients was 47 years old. There were 295 cases (67.4%) of phepchromocytoma and 143 cases (32.6%) of paraganglioma. Three hundred sixty-seven (87.8%) patients had laparoscopic approach, and 55 (12.6%) patients had laparotomy; the conversion rate from laparoscopy to laparotomy was 3.7%. Eighty-seven complications occurred in 65 patients (14.8%). No death occurred in our study and transfusion (36, 8.2%) was the most common complication. The mean follow-up length was 14 months. Independent risk factors for postoperative complications included a tumor size larger than 5.6 cm (OR = 2.427, 95% CI 1.284–4.587, *P* = 0.006), laparotomy (OR 2.590, 95% CI 1.230–5.453, *P* = 0.012), conversion to laparotomy (OR = 8.384, 95% CI 2.247–31.285, *P* = 0.002), and the operation time longer than 188 min (OR = 3.709, 95% CI 1.847–7.450, *P* < 0.001).

**Conclusion:**

Complications were not uncommon after pheochromocytoma and/or paraganglioma surgery. Tumor size, surgical type, and operation time were determined as the risk factors for postoperative complications. These factors should be considered to improve perioperative management.

## Introduction

Pheochromocytoma and/or paraganglioma are rare neuroendocrine tumors. Due to the excessive secretion of catecholamines, the main clinical symptoms include hypertension, palpitation, tachycardia, headache, and palpitations. Comprehensive preoperative evaluation and adequate anti-hypertensive treatment to restore blood volume are essential to decrease intraoperative hemodynamic instability and perioperative mortality and morbidity.

Surgical resection is still the primary strategy for the treatment of pheochromocytoma and/or paraganglioma. The surgical mortality rate had been reported as high as 50% in the past, and this rate had significantly decreased since the improvement in perioperative management and surgical techniques (1). However, pheochromocytoma and/or paraganglioma are still potential challenges to anesthesiologists.

Previous reports had focused on the incidence and risk factors of intraoperative hemodynamic instability and cardiovascular related complications (2–4). The incidence of postoperative complications after pheochromocytoma surgery ranged from 11.4 to 29.8%, and comorbidities, tumor size, catecholamine level, and surgical approach had been reported to be associated with higher risk of postoperative complications (3–5). However, few studies had evaluated the severity of postoperative complications, and the results were inconsistent. Identifying risk factors for complications may lead to improved perioperative management.

The aim of this study was to explore the relationship between patients’, tumor’s, and surgical factors and postoperative complications in patients with pheochromocytoma and/or paraganglioma.

## Materials and methods

After the approval of the Institutional Review Board, a retrospective, observational, and single-center study was conducted at Peking Union Medical College Hospital. All medical records (which included patients progress note and nurse log) of patients who had pheochromocytoma and/or paraganglioma resection at Peking Union Medical College Hospital from January 2014 to December 2019 were reviewed.

The exclusion criteria included incidental pheochromocytoma or paraganglioma without anti-hypertension medication preoperatively, pregnancy, incomplete perioperative data, patients with bilateral pheochromocytoma, paraganglioma outside abdominal (e.g., carotid body tumor, Glomus Jugular tumor, paraganglioma in the bladder, and cardiac paraganglioma), and those who had previous history of abdominal operations.

Preoperative management included selective or non-selective alpha blocker for at least 2 weeks and beta blocker for tachycardia if necessary. Perioperative target blood pressure was defined as blood pressure less than 130/80 mmHg while seated and systolic blood pressure no less than 90 mmHg while standing. All patients had operations under general anesthesia with the insertion of arterial line before induction. All operations and anesthesia were performed by qualified surgeons and anesthesiologists. Bolus infusion of phentolamine or continuous infusion of nitroprusside were administered to maintain systolic blood pressure less than 160 mmHg or no more than 30% above baseline. Continuous infusion of norepinephrine or epinephrine was administrated when systolic blood pressure less than 90 mmHg or 30% below baseline. Patients were all transferred to intensive care unit (ICU) postoperatively for further hemodynamic monitoring. Genetic analysis was performed in the majority of patients, but the results were not available for this study.

The following data were collected, which included age, sex, body mass index (BMI), comorbidities, tumor size, the level of urine catecholamine, duration of operation, intraoperative hemodynamic instability, perioperative blood product infusion, length of stay in ICU and hospital, and postoperative complications.

Deviations from the normal postoperative course recorded in medical records during hospitalization was considered as postoperative complications (6). The Clavien–Dindo classification was used to grade the severity of postoperative complication (6). Furthermore, patients who had complications with grade II or above were included in our analysis (4, 7). For patients with multiple complications, the grade was based on the most severe complication. Hemodynamic instability was defined as previously reported (8, 9). Postoperative hypotension episode was defined as SBP < 90 mmHg and/or DBP < 60 mmHg with typical clinical symptom of hypotension despite continuous infusion of norepinephrine or epinephrine.

SPSS 22.0 was used for statistical analysis. Continuous variables were presented as means ± standard deviations or medians and interquartile ranges. Category variables were presented as numbers and percentages. Student t-test or Mann–Whitney U-test was used to compare variables between two groups. Receiver Operating Characteristic Curve (ROC) was used to define cutoff point of age, tumor size, and length of operation. In addition, variables with a *P* < 0.1 were entered in binary logistic regression analysis. *P* < 0.05 was considered statistic significant.

## Results

Four hundred thirty-eight patients were included for our analysis. The median age of the patients was 47 years old [IQR 36,55], and the mean BMI was 23.9 ± 3.2. 237 patients were male (54.1%) and 201 patients were female (45.9%). Three hundred forty-five (78.8%) were ASA II, 66 (15.1%) were ASA III, and 27 (6.2%) were ASA IV at the time of the operation (see **Table 1**). There were 295 (67.1%) cases of pheochromocytoma and 143 (32.9%) cases of paraganglioma.

**Table 1:** Demographic characteristics and perioperative data of patients after resection of pheochromocytoma and/or paraganglioma.

	No complications (*n* = 373)	Complications (*n* = 65)	Total (*n* = 438)	*P*-value
Age (years, median ± IQR)	46 [35,55]	49 [42,54]	47 [36,55]	0.077
Body mass index (kg/m^2^)	23.9 ± 3.2	24.2 ± 3.0	23.9 ± 3.2	0.378
Male/female	203/170	34/31	237/201	0.752
ASA				0.633
II	295 (79.1%)	50 (76.9%)	345 (78.8%)	
III	54 (14.5%)	12 (18.5%)	66 (15.1%)	
IV	24 (6.4%)	3 (4.6%)	27 (6.2%)	
Diagnosis				0.426
Pheochromocytoma	254 (68.1%)	41 (63.1%)	295 (67.4%)	
Paraganglioma	119 (31.9%)	24 (36.9%)	143 (32.6%)	
Comorbidities				
Continuous hypertension	122 (32.7%)	18 (27.7%)	140 (32.0%)	0.424
Intermittent hypertension	108 (29.0%)	19 (29.2%)	127 (29.0%)	0.964
Diabetes mellitus	61 (16.4%)	12 (18.5%)	73 (16.7%)	0.674
Coronary artery disease	9 (2.4%)	1 (1.5%)	10 (2.3%)	0.663
Previous history of stroke	10 (2.7%)	0	10 (2.3%)	0.182
Previous history of heart failure	11 (2.9%)	3 (4.6%)	14 (3.2%)	0.481
Catecholamine-induced cardiomyopathy	22 (5.9%)	2 (3.1%)	24 (5.5%)	0.356
Pheochromocytoma crisis	10 (2.7%)	1 (1.5%)	11 (2.5%)	0.587
Preoperative management				0.255
Alpha blocker	299 (80.2%)	56 (86.2%)	355 (81.1%)	
Multiple drugs	74 (19.8%)	9 (13.8%)	83 (18.9%)	
Preoperative data				
Tumor size (cm, mean ± SD)	4.8 ± 2.0	6.9 ± 3.1	5.1 ± 2.3	0.000
Tumor necrosis	129 (34.6%)	32 (49.2%)	161 (36.8%)	0.024
Multiple tumors	28 (7.5%)	4 (6.2%)	32 (7.3%)	0.699
Urine catecholamine (median ± IQR)				
Norepinephrine (µg/24h)	101 [38.1,292.1]	83.2 [31.3,189.4]	100 [36.9,282.5]	0.106
Epinephrine (µg/24h)	3.69 [2.6,7.4]	3.64 [2.72,5.94]	3.68 [2.64,7.20]	0.803
Dopamine (µg/24h)	222.3 [170.2,316.8]	248.7 [161.4,343.6]	230.2 [169.4,322.5]	0.346
Perioperative data				
Surgery				< 0.001
Laparoscopy	333 (89.3%)	34 (52.3%)	367 (83.8%)	
Transabdominal	13 (3.5%)	0	13 (3.0%)	
Retroperitoneal	320 (85.8%)	34 (52.3%)	354 (80.8%)	
Laparotomy	36 (9.7%)	19 (29.2%)	55 (12.6%)	
Conversion to laparotomy	4 (1.0%)	12 (18.5%)	16 (3.7%)	
Length of operation (minute)	105 [85,145]	190 [135,262.5]	120 [90,160]	0.000
Hemodynamic instability	67 (18%)	20 (30.8%)	87 (19.9%)	0.027
Length of stay in ICU (day, Median ± IQR)	1 [1,1]	1 [1,2]	1 [1,1]	0.872
Length of stay in hospital (day, Median ± IQR)	5 [4,7]	8 [6.5,13.5]	6 [4,7]	0.038
Follow-up after surgery (month Median ± IQR)	13 [3,38]	18 [4,37]	14 [3,37.75]	0.294

The prevalence of continuous hypertension and intermittent hypertension was 140 (32.0%) and 127 (29.0%), respectively. Twenty-four (5.5%) patients were diagnosed as catecholamine cardiomyopathy, and 11 patients (2.5%) experienced pheochromocytoma crisis before the operations. Alpha blocker was the primary antihypertension drug preoperatively. Eighty-three patients (18.9%) had multiple drugs for preoperative preparation.

The mean diameter of the tumor was 5.1 ± 2.3 cm, and 161 patients (36.8%) had tumor necrosis presented in CT. Three hundred sixty-seven (87.8%) patients had laparoscopic operation, and 55 (12.6%) patients had laparotomy; the conversion rate from laparoscopy to laparotomy was 3.7%. Eighty-seven (19.9%) patients experienced intraoperative hemodynamic instability.

Of 438 patients, 65(14.8%) patients had 87 postoperative complications with a Dindo–Clavien grade of 2 or greater. Sixty-three patients (14.4%) and 15 patients (3.4%) had postoperative complications of grade II and grade III, respectively. Moreover, nine patients (2.1%) suffered complications of grade IV. The details of complications were presented in **Table 2**. Transfusion (36, 8.2%) was the most common complications. Eight patients (1.8%) developed postoperative hypotension. Eight (1.8%) patients had reoperation, and no death occurred in our study.

**Table 2:** Postoperative complications after surgery in patients with pheochromocytoma and paraganglioma.

Complication	Number (%)
II	63 (14.4%)
Perioperative transfusion	36 (8.2%)
Pneumonia	6 (1.4%)
Bacteremia	5 (1.1%)
Wound infection	4 (0.9%)
Adrenal insufficiency	1 (0.2%)
Arrthymia	1 (0.2%)
lleus	3 (0.7%)
DVT	4 (0.9%)
Others	3 (0.7%)
III	15 (3.4%)
Reoperation	8 (1.8%)
Pancreatic fistula	6 (1.4%)
Gastrointestinal bleeding	1 (0.2%)
IV	9 (2.1%)
Renal failure requiring hemodialysis	1 (0.2%)
Postoperative hypotension episode	8 (1.8%)

In order to explore whether there was any difference between patients with pheochromocytoma and paraganglioma, demographic characteristics and perioperative data were compared and presented in **Table 3**. There was no significant difference in age (*P* = 0.532), BMI (*P* = 0.050), sex distribution (*P* = 0.592), ASA status (*P* = 0.986). Comorbidities were similar in both groups, except that a higher incidence of previous history of heart failure (*P* = 0.010) in patients with paraganglioma. Tumor size (*P* = 0.325) and the proportion of multiple tumors (*P* = 0.318) were similar. Patients in paraganglioma were more likely to have operation in laparotomy, and the conversion rate from laparoscopy to laparotomy was higher (*P* < 0.001). Moreover, for laparoscopic approach, patients with paraganglioma were more likely to have transabdominal approach. Patients with paragangliomas had longer operation time (*P* < 0.001) and higher incidence of postoperative complications (16.8% *vs.* 13.9%, *P* = 0.426).

**Table 3:** Comparisons of demographic characteristics and perioperative data between pheochromocytoma and paraganglioma.

	Pheochromocytoma (*n* = 295)	Paraganglioma (*n* = 143)	*P*
Age (years, median ± IQR)	47 [36,55]	47 [37,56]	0.532
BMI (kg/m^2^)	23.7 ± 3.2	24.3 ± 3.1	0.050
Male/female	157/138	80/63	0.592
ASA			0.986
II	232 (78.6%)	113 (79.0%)	
III	45 (15.3%)	21 (14.7%)	
IV	18 (6.1%)	9 (6.3%)	
Comorbidities			
Continuous hypertension	89 (30.2%)	51 (35.7%)	0.248
Intermittent hypertension	86 (29.2%)	41 (28.7%)	0.917
Diabetes mellitus	50 (16.9%)	23 (16.1%)	0.820
Coronary artery disease	8 (2.7%)	2 (1.4%)	0.388
Previous history of stroke	6 (2.0%)	4 (2.8%)	0.616
Previous history of heart failure	5 (1.7%)	9 (6.3%)	0.010
Catecholamine-induced cardiomyopathy	17 (5.8%)	7 (4.9%)	0.708
Pheochromocytoma crisis	6 (2.0%)	5 (3.5%)	0.359
Preoperative management			0.310
Alpha blocker	243 (82.4%)	112 (78.3%)	
Multiple drugs	52 (17.6%)	31 (21.7%)	
Preoperative data			
Tumor size (cm, mean ± SD)	5.0 ± 2.4	5.3 ± 2.2	0.325
Multiple tumors	19 (6.4%)	13 (9.1%)	0.318
Tumor necrosis	104 (35.3%)	57 (39.9%)	0.348
Urine catecholamine (median + IQR)			
Norepinephrine (µg/24h)	97.2 [37.0,273.6]	103.5 [36.8,292.7]	0.441
Epinephrine (µg/24h)	3.91 [2.68,19.43]	3.48 [2.51,4.68]	0.011
Dopamine (µg/24h)	237.5 [171.4,322.8]	215.4 [165.9,321.4]	0.179
Surgery			< 0.001
Laparoscopy	268 (90.8%)	99 (69.2%)	0.026
Transabdominal approach	6 (2.0%)	7 (4.9%)	
Retroperitoneal approach	262 (88.8%)	92 (67.1%)	
Laparotomy	20 (6.8%)	35 (24.5%)	
Conversion to laparotomy	7 (2.4%)	9 (6.3%)	
Length of operation (minute)	105 [80,140]	140 [105,196.25]	< 0.001
Hemodynamic instability	63 (21.4%)	24 (16.8%)	0.261
Length of stay in ICU (day, median ± IQR)	1 [1,1]	1 [1,1]	0.430
Length of stay in hospital (day, median ± IQR)	5 [4,7]	7 [4,8]	0.002
Postoperative complications	41 (13.9%)	24 (16.8%)	0.426

There was no significant difference in age (*P* = 0.077), gender distribution (*P* = 0.752), BMI (*P* = 0.378), and preoperative comorbidities between patients with and without complications. Patients with complications had larger tumor (*P* < 0.001), and they were more likely to have laparotomy or convert to laparotomy intraoperatively (*P* < 0.001) (see **Table 1**). Longer operation time (*P* < 0.001), higher prevalence of intraoperative hemodynamic instability (*P* = 0.027), and longer stay in hospital (*P* = 0.038) were also observed in patients with complications (see **Table 1**).

Binary logistical regression showed tumor larger than 5.6 cm (OR = 2.427, 95% CI 1.284–4.587, *P* = 0.006), laparotomy (OR = 2.590, 95% CI 1.230–5.453, *P* = 0.012), conversion to laparotomy (OR = 8.384, 95% CI 2.247–31.285, *P* = 0.002), and the operation time longer than 188 min (OR = 3.709, 95% CI 1.847–7.450, *P* < 0.001) were risk factors of postoperative complications after resection of pheochromocytoma and paraganglioma (see **Table 4**).

**Table 4:** Multivariable analysis of risk factor associated with postoperative complications in patients with pheochromocytoma and or paraganglioma.

Factors	Odds ratio	95% CI	*P*-value
Age > 45 years old	1.470	0.482–4.487	0.498
Tumor size > 5.6 cm	2.427	1.284–4.587	0.006
Length of operation > 188 min	3.709	1.847–7.450	< 0.001
Operation type			
Laparotomy	2.590	1.230–5.453	0.012
Conversion to laparotomy	8.384	2.247–31.285	0.002
Hemodynamic instability	1.688	0.855–3.333	0.132

## Discussion

The incidence of postoperative complication in this high-volume, single-institution analysis was 14.8%, which was similar to that described in previous reports (5, 6). No death occurred in our analysis, and our results suggested the overall safety of perioperative management of patients with pheochromocytoma and or paraganglioma in our center.

Transfusion was the most common postoperative complication in our cohort, which was in accordance with reported series from other single-center study (7). Patients with pheochromocytoma and paraganglioma were at high risk of intraoperative blood loss due the dense vascular network (10). Cardiovascular morbidity, which included prolonged hypotension had been reported as the most common postoperative complication in other series (11–14). In our study, cardiovascular complications were lower than previously reported. The incidence of postoperative hypotension episode was 1.8% in our series. Moreover, Lan reported that only one patient was diagnosed with cardiac infarction in 350 patients in our center (15). The possible reasons were as followings: first, the mean age of our study was younger than previous reports. Second, our center was the high-volume referral center for pheochromocytoma and/or paraganglioma. Timely diagnosis and effective preoperative medical preparation all contribute to better outcome. Third, different definitions of complications, and the decades of the studies performed might lead to the different incidences of complications.

Tumor size had been confirmed as the risk factor for postoperative complications after laparoscopic adrenalectomy (7) and pheochromocytoma surgery. Tumor size was also demonstrated as the risk factor for intraoperative complications (8, 16, 17). Larger tumor is associated with more intraoperative blood loss (11) and higher level of catecholamine. Operation duration had also been confirmed as the risk factor of postoperative outcome in different types of surgery (18–21). Longer operation time is associated with more tissue damage and more blood loss; it also indicates the extent and complexity of the operation. Preoperative surgical plan and intraoperative cooperation between surgeons and anesthesiologists are essential to improve patients’ outcome.

Surgical type was the risk factors of postoperative complications for patients with pheochromocytoma and/or paraganglioma. Conversion to open laparotomy was reported in 16 patients. Open surgery or conversion to open surgery increased the incidence of complications by 2.590- and 8.384-folds, respectively. Open surgery or conversion to open surgery usually suggests the difficulty of tumor resection, longer operation time, more tissue injury, and more intraoperative blood loss. Although laparoscopic approach had been confirmed as the safety procedure for adrenalectomy, even for large tumors (22), the decision of surgical type should be determined based on surgeons’ experience, the comorbidities of patients, tumor size, and vicinity to blood vessels (23). Moreover, intraoperative conversion to open approach should not be hesitating when life-threatening bleeding occurs or malignancy was suspected. Although patients with paraganglioma were more likely to have open or conversion to open surgery, and the operation time was longer for paraganglioma resection, paraganglioma was not the risk factor of postoperative complications. Thus, the limited number of patients with paraganglioma and the impossibility of including all factors related to postoperative complications may contribute to this result.

This is a large study to evaluate postoperative outcome in patients with pheochromocytoma and/or paraganglioma; however, there are several limitations. First, this is a single-center analysis and selection bias could not be avoided. Second, postoperative complications were difficult to qualified due to the variety of definition, incomplete data, and lack of audit system. The incidence of postoperative complications may be underestimated due to the nature of retrospective study and incomplete date. Third, we did not include all potential parameters (e.g., dosage and duration of preoperative medication, the duration of hypertension), which may influence postoperative complications. Furthermore, we only analyzed the in-hospital complications, and long-term morbidity (e.g., 30-day readmission) was not performed.

In conclusion, postoperative complications after pheochromocytoma or paraganglioma were not uncommon. Tumor size, the type of surgery, and the duration of operation were risk factors for postoperative complications.

## Data availability statement

The raw data supporting the conclusions of this article will be made available on reasonable request and with the permission of institution where the data were generated.

## Ethics statement

The studies involving human participants were reviewed and approved by Peking Union Medical College Hospital. Written informed consent for participation was not required for this study in accordance with the national legislation and the institutional requirements.

## Author contributions

LM collected the data and wrote the manuscript. XY designed the study, performed the statistical analysis and revised the manuscript. YH supervised the study. All authors contributed to the article and approved the submitted version.
